# Perception from students regarding online synchronous interactive teaching in the clinical year during COVID-19 pandemic

**DOI:** 10.1186/s12909-022-03958-8

**Published:** 2023-01-05

**Authors:** Billy H. H. Cheung, Dominic C. C. Foo, Kent Man Chu, Michael Co, Lok Sze Lee

**Affiliations:** 1 Department of Surgery, Tung Wah Hospital, Hong Kong, SAR China; 2 Department of Surgery, LKS Faculty of Medicine, The University of Hong Kong, 2/F, Professorial Block, Queen Mary Hospital, 102 Pokfulam Road, Hong Kong, SAR China; 3 LKS Faculty of Medicine, The University of Hong Kong, Hong Kong, SAR China

**Keywords:** Online teaching, Online synchronous education, Medical education, Undergraduate education

## Abstract

**Aim:**

The global pandemic of COVID-19 has led to extensive practice of online learning. Our main objective is to compare different online synchronous interactive learning activities to evaluate students’ perceptions. Moreover, we also aim to identify factors influencing their perceptions in these classes.

**Methods:**

A cross-sectional, questionnaire-based study focusing on clinical year medical students’ perceptions and feedback was conducted between February 2021 –June 2021 at the University of Hong Kong. Online learning activities were divided into bedside teaching, practical skill session, problem-based learning (PBL) or tutorial, and lecture. A questionnaire based on the Dundee Ready Education Environment Measure (DREEM) was distributed to 716 clinical year students to document their perceptions.

**Results:**

One hundred responses were received with a response rate of 15.4% (110/716, including 96 from bedside teaching, 67 from practical skill session, 104 from PBL/tutorial, and 101 from lecture).

For the mean score of the DREEM-extracted questionnaire, online PBL/tutorial scored the highest (2.72 ± 0.54), while bedside scored the lowest (2.38 ± 0.68, *p* = 0.001). Meanwhile, there was no significant difference when we compared different school years (*p* = 0.39), age (*p* = 0.37), gender (*p* = 1.00), year of internet experience (<17 vs ≥17 years *p* = 0.59), or prior online class experience (*p* = 0.62).

When asked about students’ preference for online vs face-to-face classes. Students showed higher preferences for online PBL/tutorial (2.06 ± 0.75) and lectures (2.27 ± 0.81). Distraction remains a significant problem across all four learning activities.

A multivariate analysis was performed regarding students’ reported behavior in comparison with their perception through the DREEM-extracted questionnaire. The results showed that good audio and video quality had a significant and positive correlation with their perception of online bedside teaching, practical skill sessions, and PBL/tutorial. It also showed that the use of the video camera correlated with an increase in perception scores for lectures.

**Conclusion:**

The present analysis has demonstrated that students’ perception of different online synchronous interactive learning activities varies. Further investigations are required on minimizing distraction during online classes.

**Supplementary Information:**

The online version contains supplementary material available at 10.1186/s12909-022-03958-8.

## Introduction

The spread of the SARS-CoV-2 virus worldwide has led to a global pandemic since 2020 [1]. A broad practice of restricted mobility and social distancing has been advocated to minimize the spread of the disease [2]. Undoubtedly, the clinical areas harbor the highest risk of infection – the 2003 severe atypical respiratory syndrome (SARS) has taught us a hard lesson where a group of medical students acquired the disease after a clinical visit with a later-confirmed patient [3].

However, clinical attachment years are among the most crucial periods for medical students. Under normal circumstances, the hospital creates a comprehensive learning environment for the students – following ward rounds and various work routines with their tutors, students can learn through role-modeling; by shouldering some work in the ward, they can learn through apprenticeship; through participation in various operations, they can learn through deliberative practice; with case discussion with clinicians, they learn through scaffolding [4].

In the previous decade, e-learning has been advocated to eliminate geographical constraints and allow multimedia use. With technological advances and improved internet setup, synchronous high-quality live stream teaching has become feasible.

Several recently published short reports described new teaching tools [5], modifications in the curriculum [6, 7], and unique arrangements in teaching format [8] during this pandemic. However, there is still a limited understanding of their effectiveness, drawbacks, and student feedback. Moreover, this is the first time with a complete application of online teaching in all activities, and a comparison between these different modes of education is crucial but lacking.

Students have different perceptions among various online synchronous interactive activities. The strengths, weaknesses, and limitations of each module are best addressed individually to deliver a more in-depth understanding. With around a year of adoption, most online learning activities have been optimized, making it suitable to evaluate students’ perceptions and satisfaction to improve the current teaching system.

The main objective of the current study is to assess the perception of medical students regarding online synchronous interactive teaching in the clinical year during COVID-19 pandemic. Moreover, we also aim to identify factors influencing their perception of these activities. All this information is aimed at guiding us for recommendations for future online classes.

## Methods

The current study is a cross-sectional, questionnaire-based study focusing on clinical year medical students’ perceptions and feedback among various online synchronous interactive learning activities.

### Learning activities

We divided the online synchronous learning activities into four major types – bedside teaching, where authentic patients were involved; practical skill sessions, where clinical skills, such as suturing, catheter insertion, and clinical examinations, were taught; small group tutorials or problem-based learning; and lectures. Different modes of online synchronous learning activities were evaluated separately.

Various adoptions had been carried out at the University of Hong Kong. “Webside teaching”, coined by Tsang et al. [9], comprises interactive video conference technology with patients utilizing high-definition video cameras and microphones. Co et al. [10] used a multi-camera setup, in particular one focusing on his hand movement, for basic surgical skill classes. In ophthalmology, Shih et al. [11] introduced some video-based and written materials to precede and complement Zoom platform-based small group tutorials.

### Questionnaire design

The first part explored students’ backgrounds, including their experience of internet use and previous online learning experience (Additional file 1: Appendix 1).

The second to fourth part of questionnaire was divided into the four modes of online teaching. At the second part, questions regarding the situation and circumstances during class were documented. Further questions were asked focusing on the student’s behavior during class with the Likert scale, ranging from “strongly disagree” to “strongly agree”. The rating was transferred into marks, with “strongly agree” being four and “strongly disagree” being zero.

At the third part, the focus was on perceptions about students’ learning, which was shown to correlate with their academic performance, learning pleasure, and propensity to achieve learning outcomes [12]. This part of the questionnaire was extracted from the well-validated Dundee Ready Education Environment Measure (DREEM) for medical education environments evaluation [13, 14]. The original DREEM questionnaire composes of 50 questions divided into five different aspects - Students’ perceptions of learning (POL) (12 items); Students’ perceptions of teachers (POT) (11 items); Students’ academic self-perceptions (ASP) (8 items); Students’ perceptions of atmosphere (POA) (12 items); and Students’ social self-perceptions (SSP) (7 items) [13]. After reviewing the original questionnaire, 17 more relevant questions were chosen with mutual agreement in our research team to keep the questionnaire concise yet representative - Five questions in the POL category, three questions in the POT category, three questions in the ASP category, and seven in the POA category. No question from the SSP category was chosen as this area is less relevant to the learning process that we were focusing on. This arrangement provides a broader range of feedback from our students, which offers more insight than a previous study by Dost et al. [15]. The mean score was calculated to compare individual items. A mean score of 3.5 is regarded as exceptionally strong areas, whereas items with a mean score of 2.0 need particular attention; items with mean scores between 2 and 3 are areas that could be improved [16].

Because of a lack of control, the fourth part was dedicated to a comparison between online and face-to-face activities. The last part was two open-ended questions regarding their online experience and recommendations.

### Study period

The study period was approximately one year after the first confirmed COVID-19 case in Hong Kong [17]. The current research was a prospective study conducted between February 2021 –June 2021 at the Li Ka Shing Faculty of Medicine, University of Hong Kong.

### Participants and ethical considerations

The subjects were medical students in their clinical year (716 year 4–6 students). A comprehensive list of their official emails was obtained from the faculty office, and an open invitation was sent to these email addresses. The survey was created using Jotform (https://form.jotform.com/210532006871446), an online surveying software (2021, San Francisco, US [18]). All data collected was non-identifiable and used for research purposes only. A mandatory selection box consenting to participation at the beginning of the survey was provided on the first page of the questionnaire in both the online and paper format, ensuring a 100% consent rate. Students were also invited to participate in the study after class to increase the response rate. This was only done after performance rating to ensure genuine voluntary participation, and teachers had confirmed the absence of prior participation in this study before distributing the invitation link. The current study was approved by the Faculty Research Ethics Committee of the Faculty of Education.

### Data analysis

Data were extracted into Excel (Excel V.16.29, 2019) from Jotform questionnaires. Then they were re-coded and transferred into the statistical package for social sciences SPSS version 22.0.0 (IBM SPSS, IBM Corporation, Armonk, NY). The demographic data and their perceptions were described.

To facilitate subsequent comparison, students were further categorized into two groups – yes, neutral, or no, for their behavior and experience during class. Descriptive statistics (percentages, mean, and standard errors of the mean) were used to describe the quantitative variables. At the same time, analysis of variance (ANOVA) was used to compare the mean score between the four principal learning activities. In each learning activity, Student’s t-test was used to indicate the difference between factors potentially influencing the students’ perception. Multivariate logistic regression analysis was then performed to indicate any correlations between these factors and the scores and to calculate the Odds ratio. Results are considered statistically significant when *p* < 0.05.

For the open-ended questions, results were summarized to reflect their general impression. Insightful comments, concerns, or suggestions were highlighted.

## Results

### Cohort demographics

**Fig. 1:**
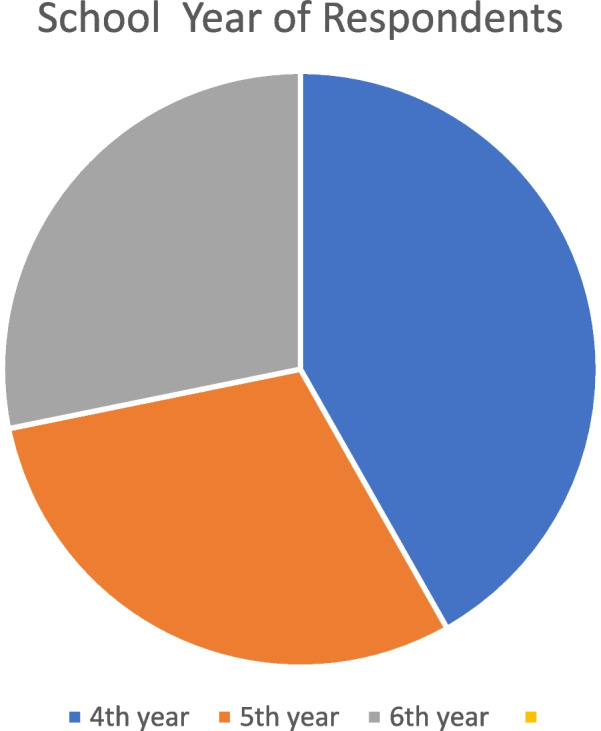
Distribution of Respondents from Various School Years

In the study period, among the 716 clinical year medical students, 110 replies were received, with a response rate of 15.4% (110/716). Of the 110 responses collected, 45.5% (n = 50) of respondents were women and 54.5% (n = 60) were men. 41.8% (n = 46) were from the fourth year (first year of their three-year clinical clerkship), 30.0% (n = 33) were from the fifth year, and 28.2% (n = 31) were from the sixth year (Fig. 1). The mean age of our respondents is 23.0 (range 21–30), with the mean year of internet use 15.9 years (range 10–23). Eighty-nine of them (80.9%) had prior online learning experiences before the COVID-19 period.

**Fig. 2:**
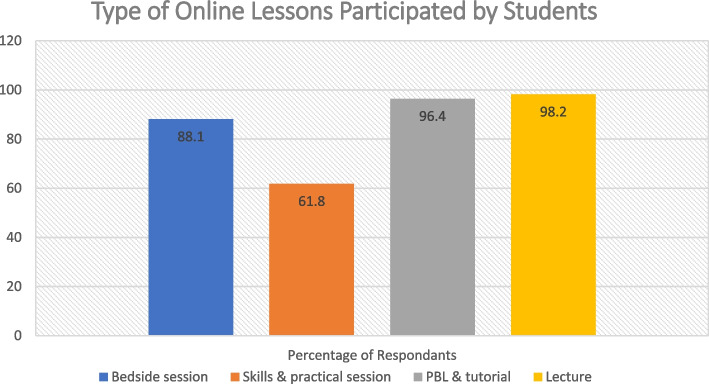
Type of Online Lessons Participated by Students

Regarding the type of lesson they experienced, most had had online bedside teaching (89.1%, n = 98). Slightly fewer students had online practical and skills lesion during this period (61.8%, 68/110), and almost all of them experienced online PBL and tutorials (96.4%, n = 106) and lectures (98.2%, n = 108) (Fig. 2). This makes up a total of three hundred sixty-eight valid responses with all four learning activities combined.

### Students’ behavior and perception during class, the DREEM-extracted questionnaire, and their preference toward online classes

#### Learning circumstances and students’ behavior during class

Focusing on their experience, 44.8% (165/368) of them reported spending less than two hours on online classes in the previous four weeks. A vast majority of them (89.1%, 328/368) underwent the lesson at home, and 89.9% (331/368) reported using a computer for the class. For their behavior, most of them were muted (79.6%, 293/368), but 63.6% of them (234/368) switched on the video during class. Regarding the technical aspect, the results looked satisfying, with 76.9% of them (283/368) agreed that the audio and video were good, and 83.4% (307/368) agreed that the material was clearly shown. The software was reported to be easy to use in 87.5% of them (332/368).

#### Overall mean score from the DREEM-extracted questionnaire

**Fig. 3:**
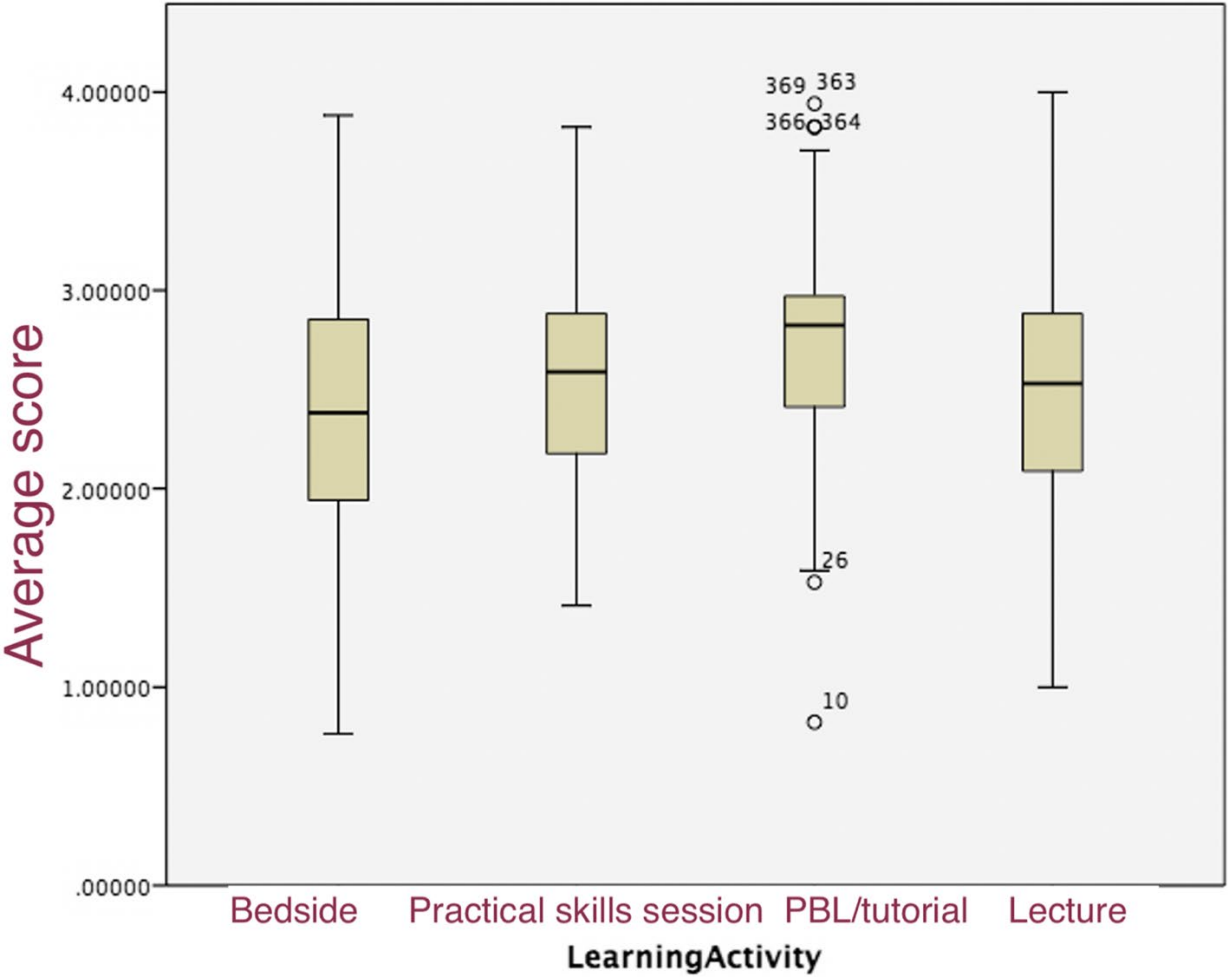
Overall mean scores of the DREEM-extracted questionnaire

The overall mean scores of the DREEM-extracted questionnaire comparing the four learning activities are summarised in Fig. 3. We can see that the mean score is the highest for the PBL/tutorial group and the worst for the bedside group (*p* = 0.001). Meanwhile, there was no significant difference when we compared different school years (*p* = 0.39), age (*p* = 0.37), gender (*p* = 1.00), year of internet experience (<17 vs ≥17 years p = 0.59), or prior online class experience (*p* = 0.62).

The detailed results for the DREEM-extracted questionnaire are laid out in Table 1. There were two items with a mean score below 2.0 – that include question 1 in the online bedside teaching group (1.61 ± 0.81, boldened), which is also the lowest score item, and question 14 among the practical skills sessions group (1.87 ± 0.92, boldened). For the overall mean score, question 14 is the lowest score item among the 14 questions (2.06 ± 0.99, row No. 7). We could not identify any modifiable factors contributing to the low score. On the other hand, question 7 scores the highest (2.97 ± 0.75, row No. 14) with two subgroups scores above 3 (bedside teaching group, mean score 3.02 ± 0.78; PBL/tutorial group, mean score 3.05 ± 0.73, both underlined).

**Table 1:** Responses to DREEM-extracted questionnaire items among the four learning activities

	Bedside teaching	Practical skills session	PBL/tutorial	Lecture	Overall
Perception of learning					
1. I was encouraged to participate in class	**1.61 ± 0.81**	2.73 ± 0.64	2.99 ± 0.70	2.23 ± 1.01	2.38 ± 0.98
2. The teaching was sufficiently concerned to develop my competence	2.47 ± 0.95	2.55 ± 0.78	2.85 ± 0.75	2.64 ± 0.80	2.64 ± 0.83
3. The teaching was sufficiently concerned to develop my confidence	2.30 ± 1.00	2.56 ± 0.88	2.82 ± 0.80	2.58 ± 0.83	2.57 ± 0.89
4. The teaching time was put to good use	2.52 ± 0.94	2.58 ± 0.80	2.79 ± 0.80	2.79 ± 0.74	2.68 ± 0.83
5. The teaching encouraged me to be an active learner	2.42 ± 0.97	2.69 ± 0.78	2.75 ± 0.83	2.49 ± 0.90	2.58 ± 0.89
Students’ perceptions of teachers					
6. The teachers were good at providing feedback to students	2.50 ± 0.94	2.82 ± 0.65	2.89 ± 0.75	2.40 ± 0.92	2.64 ± 0.86
7. The teachers were well prepared for their classes	3.02 ± 0.78	2.88 ± 0.75	3.05 ± 0.73	2.91 ± 0.73	2.97 ± 0.75
Students’ academic self-perceptions					
8. I am confident about passing this year	2.14 ± 1.05	2.40 ± 0.97	2.40 ± 0.95	2.37 ± 1.00	2.32 ± 1.00
9. I feel I am being well prepared for my profession	2.18 ± 1.00	2.24 ± 0.97	2.45 ± 0.91	2.45 ± 0.91	2.34 ± 0.95
10. My problem-solving skills were being well developed here	2.41 ± 0.96	2.60 ± 0.87	2.69 ± 0.77	2.37 ± 0.97	2.51 ± 0.90
Students’ perceptions of the atmosphere					
11. The atmosphere was relaxed	2.66 ± 0.89	2.88 ± 0.69	2.85 ± 0.77	2.88 ± 0.80	2.81 ± 0.80
12. There were opportunities for me to develop interpersonal skills	2.19 ± 1.03	2.45 ± 0.88	2.59 ± 0.82	2.19 ± 1.09	2.35 ± 0.98
13. I felt comfortable in class socially	2.55 ± 0.91	2.64 ± 0.81	2.81 ± 0.72	2.65 ± 0.85	2.67 ± 0.82
14. I found the experience disappointing^a^	2.10 ± 0.95	**1.87 ± 0.92**	2.15 ± 1.02	2.05 ± 1.05	2.06 ± 0.99
15. I was able to concentrate well	2.44 ± 0.89	2.61 ± 0.74	2.55 ± 0.89	2.50 ± 1.03	2.52 ± 0.91
16. The atmosphere motivated me as a learner	2.24 ± 0.98	2.47 ± 0.86	2.67 ± 0.85	2.48 ± 0.88	2.47 ± 0.91
17. I felt able to ask the questions I want	2.68 ± 0.87	2.77 ± 0.67	2.92 ± 0.67	2.62 ± 0.83	2.75 ± 0.78
18. Overall mean scores	2.38 ± 0.68	2.57 ± 0.52	2.72 ± 0.54	2.51 ± 0.59	2.55 ± 0.60

^a^Reverse scoring

#### Comparison between online and face-to-face classes

Students were asked to compare the online class to the face-to-face counterpart (Table 2). In all four aspects of the comparisons, online bedside teaching was inferior to face-to-face, with all of them scoring below 2.0. This means that students are more preferred towards face-to-face classes in this category. On the other hand, online PBL/tutorial and lecture are mostly preferred by students (Table 2, row No. 4) and the overall mean scores (Table 2, row No. 5) in this section. Students indicated that they were prone to distraction among all four learning activities (Table 2, Bolded), worst in the bedside teaching group. None of the items scored above 3.0, and the best mean scores were achieved in the online lecture group with a significantly higher mean score of 2.27 ± 0.81 (*p* = 0.000).

**Table 2:** Students’ preference toward online classes among the four learning activities

	Bedside teaching	Practical skills session	PBL/tutorial	Lecture	Overall
Compared to face-to-face class:					
1. My participation was better online.	1.94 ± 1.04	2.00 ± 1.03	2.15 ± 0.96	2.33 ± 1.11	2.11 ± 1.05
**2. I was more prone to distraction.**^**a**^	**1.40 ± 0.96**	**1.61 ± 0.92**	**1.51 ± 0.92**	**1.52 ± 0.93**	**1.50 ± 0.93**
3. I learned more efficiently online.	1.99 ± 1.06	2.03 ± 1.02	2.30 ± 0.91	2.62 ± 1.08	2.25 ± 1.04
4. In the future, I prefer online classes than face-to-face classes	1.84 ± 1.24	1.99 ± 1.19	2.27 ± 1.15	2.62 ± 1.14	2.20 ± 1.21
5. Overall mean score	1.79 ± 0.81	1.83 ± 0.79	2.06 ± 0.75	2.27 ± 0.81	2.00 ± 0.81

^a^Reverse scoring

### Evaluation of individual learning activity

We tried to evaluate the students’ behavior for individual learning activities and then compare it to the overall mean of the DREEM-extracted questionnaire scores and the preference for online classes. The demographic factors showed no correlation to their scores in all four activities. For the online practical skills session, students who used smartphones/tablets for the class and students who spent more than two hours in the past four weeks rated a higher score in this part of the questionnaire (Table 3). Such difference was not demonstrated in other learning activities.

**Table 3:** Behavior during online practical skills session vs the DREEM-extracted questionnaire score

		N=	Mean score	*P* =
Hours spent in the past 4 weeks	<2 hours	39	2.46 ± 0.54	0.04
	≥2 hours	25	2.74 ± 0.50	
Device used	Computer	59	2.54 ± 0.52	0.05
	Tablet/smartphone	4	3.09 ± 0.49	
I was muted most of the time	Yes	60	2.55 ± 0.51	0.21
	No	7	2.81 ± 0.61	
The video was switched on most of the time	Agree/Strongly agreed	47	2.69 ± 0.45	0.006
	Neutral	20	2.31 ± 0.59	

Regarding students’ behavior and perception of the DREEM-extracted questionnaire score, the results showed that good audio and video quality showed a significant and positive correlation with their perception of online bedside teaching, practical skill sessions, and PBL/tutorial. It also showed that switching on the video camera correlated with an increase in perception scores for lectures after the multivariate analysis (Table 4). For the comparison with face-to-face classes, there is a negative correlation between their preference toward online learning in PBL/tutorial when students were muted most of the time (Table 5). When the video is switched on most of the time, there is a positive correlation with their preference for online learning (Table 5).

**Table 4:** Correlation between students’ behavior and their perception with the DREEM-extracted questionnaire score

	Bedside teaching	Practical skills session	PBL/tutorial	Lecture
I was muted most of the time.				
The video being switched on most of the time				0.31 (CI 0.08–0.64)
Good audio and video quality	0.26 (CI 0.03–0.74)	0.39 (CI 0.06–0.71)	0.28 (CI 0.09–0.65)

**Table 5:** Correlation between students’ behavior and their perception with their preference toward online classes

	Bedside teaching	Practical skills session	PBL/tutorial	Lecture
I was muted most of the time.			−0.23 (CI −0.64 - -0.05)	
The video being switched on most of the time				0.38 (CI 0.15–0.60)
Good audio and video quality				

### Response to the open-ended questions

Online bedside teaching, which is new in our faculty, attracted the most feedback among the four activities from our students, with 42 feedbacks among the 96 respondents (43.8%). Many only left a brief comment with a similar amount of positive and negative comments (12/42, 28.6%, respectively). Some students expressed frustration with the online bedside, especially about the student-patient interaction and the lack of physical contact. Student A: “… It lacks the human touch and interactions, making learning less fun….” Student B: “…I still feel incompetent in detecting signs….” On the positive side, some students showed their support and appreciation for the effort of their teachers, especially during the pandemic with tight social restrictions. There were twenty-three suggestions provided by our respondents. Thirteen of them (13/23, 56.5%) preferred face-to-face over online, with another two (2/23, 8.7%) picked online and commented it as “time-efficient”, and three (3/23, 13.0%) preferred a hybrid approach.

There were notably fewer responses to the online practical skill session– only eleven provided their comments out of the 68 respondents (16.2%). Three (3/11, 27.3%) mentioned that online classes could not replace face-to-face classes in this category due to the need for practice and feedback. There were only two positive detailed comments - One mentioned that it was suitable for investigation learning and discussions such as electrocardiogram and chest X-ray. Another one said that it provided an optimal view compared to face-to-face: “I can see better (not standing too distant away from the tutor, and the tutor does not have to shout for us to hear).”

Twenty-eight responses were received for the online PBL/tutorial (28/106, 26.4%). Twenty (20/28, 71.4%) of them thought it was “good”, “great”, or “suitable”. Four of them thought it was “okay”. There were no negative comments in this open session. Many comments prized online PBL/tutorial for its efficiency and the time saved, and some even showed a preference for online classes. Student C: “It is efficient. It is easier to hear everyone clearly than in a room.” Student D: “More chance to voice out my opinions and answer the questions in PBL.” On the other hand, a few preferred face-to-face due to better interaction. Student E: “…clash of voice and internet connection hinder interaction between students and tutors.” Student E: “…People tend to mute in tutorial / PBL, hence less discussion….”

For the online lecture, twenty-nine comments were obtained from our 108 respondents in the online lecture session (26.9%). Among them, nineteen (19/29, 65.5%) left some positive comments, while another two respondents commented lectures were “okay” (6.9%). Among these positive reviews, the most mentioned benefits were time-efficient and flexibility. Student F: “Watching the online lectures at a higher speed is efficient for my study….” Student G: “This is the best way to learn from lectures. I can pause the recorded video and type notes….” One respondent tried to divide lectures into two types – the interactive type and the unidirectional type. He suggested face-to-face teaching sessions for interactive lectures, especially when case scenarios involved a joint discussion.

## Discussion

### Overall

The current study provided a more detailed assessment of students’ perception of various online synchronous interactive learning activities during the COVID-19 pandemic. From our results, it is encouraging to know our students are satisfied with the preparation of our teachers for their classes, especially in online bedside teaching and problem-based learning (Table 1). However, we can also see room for improvement in many areas.

One item that scores particularly low across all four learning activities is the disappointment regarding the online learning experience (2.06 ± 0.99, Table 1). Although students commonly used online resources before the pandemic, they were primarily additional components supporting traditional face-to-face teaching rather than primary learning activities [19]. The faculty never anticipated a sudden yet complete switch to the online environment, which is understandably prone to shortcomings. An earlier survey in the UK in May 2020 explored medical students’ perceptions and found that they were not enjoying or engaged in online learning in general [15]. On the bright side, similar to this UK study, our students also acknowledged that online education is time-efficient and flexible.

When we compare online to face-to-face classes, distraction is the main issue expressed by our respondents (Table 2). However, there was no association with any modifiable factors in our study. There was no significant correlation between their perception with any demographic factor, learning experience, or situation during the class.

### Online bedside

The online version is a new learning activity for our students, primarily developed and evolved since the pandemic. It scored the lowest on the DREEM-extracted questionnaire and the preference session (Tables 1 and 2). The lowest score was seen in the item “I was encouraged to participate in class” with a mean score of 1.61 ± 0.81. A large-scale qualitative analysis focusing on obstacles during online learning showed that social interaction correlates with enjoyment, effectiveness, and willingness to continue online learning [20]. As suggested by some students, a straightforward strategy is to divide the class into smaller groups to receive more attention from their teacher.

The score in the DREEM-extracted questionnaire is associated with audio and video quality (Table 3). This result indicates the importance of high-speed internet infrastructure and adequate training to educators to ensure quality. The current software and hardware provide explicit videos and audio. However, when they were not correctly set up or used, the learning experience could be disrupted. This result also communicates an important message to the educators to monitor the quality during class and seek help if any poor internet signal is encountered. This can affect students’ perception and potentially the learning outcome. There was no factor identified associated with the low preference for online bedside. However, a few potential barriers could be seen from our open comment session, including difficulty building rapport with the patient, lack of human touch, and a lack of tactile feedback for physical signs.

From earlier studies, inadequate clinical examination carries the potential for subsequent medical error [21], and physical examination was found to have a substantial effect on the management of patients [22, 23]. To replace physical examination on actual patients, simulation training has been applied for teaching physical examination [24, 25]. A meta-analysis was performed to examine simulation training for breast and pelvic physical examinations. They concluded that simulation training is associated with positive effects on skills outcomes compared to no intervention. Unfortunately, simulation training is associated with a lack of authenticity, possible higher cost, and availability of specific models, as most simulation models are task-specific rather than comprehensive [26]. On top of this, bedside teaching is not only a skill-training process. Since the class is commonly taught in clinical areas with an actual patient under some active management, this creates a comprehensive learning experience applying multiple learning theories, including social learning, behaviorism, constructivism, and the cognitive apprenticeship mode [27]. Again, this may not be easily carried out on an online platform since students would be staying at a remote place facing a screen instead of embracing a clinical environment.

### Online practical skill session

The online version is another new form of teaching developed during the current pandemic. Both surgical and clinical examination skills not involving patient was conducted online during this period. Multiple modifications have been suggested to facilitate remote skill teaching while students could be staying at home safely during classes [10, 28–33]. Some of these modifications involve a creative approach to adapt to resource limitations. Some online courses require students to utilize household applications and other readily available resources to mimic surgical tools [30, 32] while others through the distribution of mini-practical kids [10, 33]. One of them performed a comparative study with a 2019 face-to-face cohort with a superior result with an earlier proficiency in suturing and knot-tying skills with less coaching [31].

From our results, online practical skills score the second-highest in the DREEM-extracted questionnaire, just inferior to online PBL (Table 1). This result reflects a relatively positive perception of the four online learning activities. Like bedside teaching, clear video and audio are associated with a higher score. One possible explanation is that because students need to observe the demonstrations, practice, and perform return demonstrations in front of the camera, a clear video and audio allow for a more detailed observation with better assessment and guidance.

On the other hand, this should not be overlooked because there is still a low preference for online practical skill sessions compared to face-to-face sessions (Table 2). Despite the positive results reported for online practical skills sessions [31, 32], most other studies only compare pre-and post-intervention skills proficiency but not face-to-face teaching [28, 30, 33]. Besides, the online class material is not standardized, and hence the external validity of these studies may be limited.

#### Online PBL and tutorial

Online PBL achieved the highest mean score from the DREEM-extracted questionnaire (2.72 ± 0.54, Table 4). Studies comparing online and face-to-face PBL have been performed for more than a decade with reliable results [34–37]. Their results indicated that it is feasible to conduct PBL online with an enhancement of their ability of critical thinking and fulfilled the intended learning objectives. In agreement with these results, online PBL could enhance metacognitive skills, problem-solving ability, and teamwork. Compatibly, in our study, students were positive regarding the online PBL, praising its efficiency, and some even became more involved.

From our results, clear video and audio are associated with a higher DREEM-extracted score, and students who muted most of the time have a lesser preference for online. Similar to the results in online bedside, educators should ensure their signal quality during class. In addition, educators should also encourage students to unmute themselves, given that PBL is highly interactive in which students learn through discussion and become active members, contributing to solving a clinical scenario with joint efforts [38].

Despite a higher DREEM-extracted questionnaire score, Foo et al. discovered that the scores from online PBL groups were lower than the previous face-to-face cohort [39]. They suggested that this lower score, persistent despite in the following tutorial, is potentially due to more than transitional issues. Students were more neutral regarding whether online or face-to-face PBL should be performed for future arrangements and expressed concerns regarding the distraction (Table 2).

#### Online lecture

Both synchronous and asynchronous lectures were conducted in our faculty during this period. Despite that relatively low mean score for the DREEM-extracted questionnaire (2.51 ± 0.59, Table 1), the mean score compared to face-to-face learning is ranked the highest among these four modes of learning (2.27 ± 0.81, Table 2). The majority of our respondents gave some positive feedback, and a number of them suggested continuing its use even when the pandemic is over. The most prized advantage is time efficiency, and that play-back function provides a flexible approach for a revisit. Reports indicate students’ preference towards pure synchronous online teaching over recorded videos with the concern over a negative influence on the engagement, including a letter to the Editor from Motie et al. [40]. One of the significant concerns lay in a potential decrease in real-time engagement during synchronous teaching, as there would be no consequence with reduced interaction and attention. This argument is supported by an earlier qualitative study by Dommett et al. [41]. They found that students might substitute attendance at live lectures with decreased questions. They also expressed concern that students might miss essential concepts from the pre-recorded videos as they tended to skip through them. However, there are also supports for asynchronous videos. Hsin et al. [42] described the use of video mini-lectures to improve students’ satisfaction and increase average grades with a significantly higher percentage of students and with much less instructor intervention. The ability of learners to control their learning pace with video also indicates a shift from educator-centered learning in the traditional face-to-face lecture towards a learner-based approach [43], which is potentially beneficial with an increase in learners’ autonomy. A more detailed analysis focusing on learners’ satisfaction and learning outcome found that videos compliant with multimedia learning principles are highly satisfying [44]. Despite the above conflicting evidence, asynchronous videos are possibly helpful, provided they are properly constructed while students’ attendance and engagement in class can be maintained. Currently, most lectures become pre-recorded videos, followed by synchronous online question-and-answer sessions, potentially maintaining their engagement by lack of overlapping. To further improve their satisfaction during online synchronous lectures, we also recommend that videos should be switched on based on our results from the regression analysis.

### Limitation

Despite the interesting findings in our study, the results may need to be interpreted with caution. The current study is limited to a single institute, limiting its external validity to a certain. With only a part of the DREEM questionnaire being selected, the perception assessment could be incomprehensive and cannot reflect the whole picture. In addition, the behaviors were only retrospectively reported by our students. There was no other objective measurement, such as in-class observation or recording to verify, potentially contributing to report bias. Lastly, there could be a selection bias as students who felt satisfied after class might be more willing to fill in the questionnaire.

## Conclusion

The present analysis has demonstrated that students’ perception of different online synchronous interactive learning activities varies. Continuous effort should be encouraged to maximize patient exposure for our clinical year medical students, particularly for bedside teaching. With a positive perception regarding online PBL/tutorials and a strong preference for online lectures, there is a high possibility that these classes will remain online. Good audio and video quality should be ensured. Implementation for online practical skill classes should remain cautious as it is not preferred to face-to-face classes, despite relatively high scores in the DREEM-extracted questionnaire. Moreover, further investigations are required to minimize distraction during online classes.

## Supplementary Information

**Additional file 1.** Questionnaire.

## Data Availability

Please contact Dr. Billy HH Cheung via email at bhhcheung@gmail.com if further data and materials are required.
