# Supplementary material for: Perception from students regarding online synchronous interactive teaching in the clinical year during COVID-19 pandemic
Source: BMC Med Educ. 2023 Jan 5;23:5. doi: 10.1186/s12909-022-03958-8 (PMC9812743; doi:10.1186/s12909-022-03958-8)
Supplement: Supplementary file 1 — Additional file 1. Questionnaire. [file 12909_2022_3958_MOESM1_ESM.docx]

**Perception from Students regarding Online Synchronous Interactive Teaching in the Clinical Year during COVID-19 pandemic**

**Background**

|  |  |  |  |  |  |
| --- | --- | --- | --- | --- | --- |
| 1. Your age | ______ |  |  |  |  |
| 1. Gender | Male | Female |  |  |  |
| 1. School year | ______ |  |  |  |  |
| 1. Years of experience with internet use | ______ |  |  |  |  |
| 1. Have you ever had online lesson before this COVID-19 pandemic ? | Yes |  | No |  |  |
| 1. What online learning activities have you joined during this COVID-19 pandemic? | Online bedside session | Skills and practical session | PBL/tutorial | Lecture | Others ( Please state) |
|  |  |  |  |  |  |
|  |  |  |  |  |  |

**Online bedside session/**

**Skills and practical session/**

**PBL or tutorial/**

**Lecture**

**During the class**

| 1. Where were the tutorial conducted? (choose the most relevant one) | Home | Cafe | Study room | Others:_______ |  |  |
| --- | --- | --- | --- | --- | --- | --- |
| 1. Device used for the class   (choose the most relevant one) | Desktop | Laptop | Tablet | smartphone | Others |  |
| 1. How many hours have you received in total over the past four weeks | <2 | 2-5 | 5-10 | 10-20 | >20 |  |

|  | Strongly agree | Agree | Natural | Disagree | Strongly disagree |  |
| --- | --- | --- | --- | --- | --- | --- |
| During the class |  |  |  |  |  |  |
| 1. I was muted most of the time. |  |  |  |  |  |  |
| 1. I switched on my video most of the time. |  |  |  |  |  |  |
| 1. There were other people in the same room most of the time. |  |  |  |  |  |  |
| 1. The audio and video quality were good and stable. |  |  |  |  |  |  |
| 1. The software was easy to use |  |  |  |  |  |  |
| 1. The teaching material was shown clearly on the screen. |  |  |  |  |  |  |
|  |  |  |  |  |  |  |
|  |  |  |  |  |  |  |
| **Feedback – perception** |  |  |  |  |  |  |
| 1. I am encouraged to participate in class |  |  |  |  |  |  |
| 1. The teaching is sufficiently concerned to develop my competence |  |  |  |  |  |  |
| 1. The teaching is sufficiently concerned to develop my confidence |  |  |  |  |  |  |
| 1. The teaching time is put to good use |  |  |  |  |  |  |
| 1. The teaching encourages me to be an active learner |  |  |  |  |  |  |
| 1. The teachers are good at providing feedback to students |  |  |  |  |  |  |
| 1. The teachers are well prepared for their classes |  |  |  |  |  |  |
| 1. I am confident about passing this year |  |  |  |  |  |  |
| 1. I feel I am being well prepared for my profession |  |  |  |  |  |  |
| 1. My problem-solving skills are being well developed here |  |  |  |  |  |  |
| 1. The atmosphere is relaxed |  |  |  |  |  |  |
| 1. There are opportunities for me to develop interpersonal skills |  |  |  |  |  |  |
| 1. I feel comfortable in class socially |  |  |  |  |  |  |
| 1. I find the experience disappointing |  |  |  |  |  |  |
| 1. I am able to concentrate well |  |  |  |  |  |  |
| 1. The atmosphere motivates me as a learner |  |  |  |  |  |  |
| 1. I feel able to ask the questions I want |  |  |  |  |  |  |
|  |  |  |  |  |  |  |
| Compared to face-to-face class: |  |  |  |  |  |  |
| 1. My participation was better online. |  |  |  |  |  |  |
| 1. I was more prone to distraction. |  |  |  |  |  |  |
| 1. I learned more efficiently online. |  |  |  |  |  |  |
| 1. In the future, I prefer online classes than face-to-face classes. |  |  |  |  |  |  |
|  |  |  |  |  |  |  |

I think the experience is: _____________________________

Comment for improvement:

______________________________________________________________________________________________________________________________________________________
